# Supplementary material for: Heat adaptation of phage T7 under an extended genetic code
Source: Virus Evol. 2021 Dec 1;7(2):veab100. doi: 10.1093/ve/veab100 (PMC8923235; doi:10.1093/ve/veab100)
Supplement: veab100_Supp [file veab100_supp.zip › Sequencing_Analysis.zip!/Sequencing_Analysis/S2/evidence/JC_SIDE_1_63.html]

BRESEQ :: Evidence


| New junction evidence | | | | | | | | | | | |
| --- | --- | --- | --- | --- | --- | --- | --- | --- | --- | --- | --- |
|  | | seq id | position | reads (cov) | reads (cov) | score | skew | freq | annotation | gene | product |
| \* | ? | NC\_001604.1 | = 13568 | 61 (0.010) | 36 (0.010) | 16/588 | 48.0 | 43.4% | intergenic (–/–) | *–/–* | –/– |
|  | ? | NC\_001604.1 | 14352 = | 34 (0.010) |  |  |  |  | intergenic (–/–) | *–/–* | –/– |
| Rejected: Coverage evenness skew score above cutoff. | | | | | | | | | | | |

Only 100 of 5460 total aligned reads displayed.  

`GGATTCTTGATGACTTTCCAGACGACTACGAGAAGTTTCGCTGGAGAGTCCCATTCTAATACGACTCACTAAAGGAGACACACCATGTTCAAACTGATTAAGAAGTTAGGCCAACTGCTGGTTCGTATGTACAACGTGGAAGCCAAGCGACTGAACGATGAGGCTCGTAAAGAGGCCACACAGTCACGCGCTCTGGCGATTCGCTCCAACGAACTGGCTGACAGTGCATCCACTAAAGTTACCGAGGCTGCCCGTGTGGCAAACCAAGCTCAACAGCTTTCCAAATTCTTTGAGTAATCAAACAGGAGAAACCATTATGTCTAACGTAGCTGAAACTATCCGTCTATCCGATACAGCTGACCAGTGGAACCGTCGAGTCCACATCAACGTTCGCAACGGTAAGGCGACTATGGTTTACCGCTGGAAGGACTCTAAGTCCTCTAAGAATCACACTCAGCGTATGACGTTGACAGATGAGCAAGCACTGCGTCTGGTCAATGCGCTTACCAAAGCTGCCGTGACAGCAATTCATGAAGCTGGTCGCGTCAATGAAGCTATGGCTATCCTCGACAAGATTGATAACTAAGAGTGGTAT  >  NC_001604.1/13268‑13862`  

                                                                                                                                                                                                                                                                                                            `|`                                                                                                                                                                                                                                                                                                        
  `aTTCTTGATGACTTTCCAGACGACTACGAGAAGTTTCGCTGGAGACTCCCATTCTAATACGACTCACTAAAGGAGACACACCATGTTCAAACTGATTAAGAAGTTAGGCCAACTGCTGGTTCGTATGTACAACGTGGAAGCCAAGCGACTGAACGATGAGGCTCGTAAAGAGGCCACACAGTCACGCGCTCTGGCGATTCGCTCCAACGAACTGGATGACAGTGCATCCACTAAAGTTACCGAGGCTGCCCGTGTGGCAAACCAAGCTCAACAGCTTTCCAAATTCTTTGAGTAATCaata                                                                                                                                                                                                                                                                                                      <  1:100086/301‑3 (MQ=255)`  
     `cTTGATGACTTTCCAGACGACTACGAGAAGTTTCGCTGGAGAGTCCCATTCTAATACGACTCACTAAAGGAGACACACCATGTTCAAACTGATTAAGAAGTTAGGCCAACTGCTGGTTCGTATGTACAACGTGGAAGCCAAGCGACTGAACGATGAGGCTCGTAAAGAGGCCACACAGTCACGCGCTCTGGCGATTCGCTCCAACGAACTGGCTGACAGTGCATCCACTAAAGTTACCGAGGCTGCCCGTGTGGCAAACCAAGCTCAACAGCTTTCCAAATTCTTTGAGTAATCACACAGG                                                                                                                                                                                                                                                                                                   >  1:354102‑M1/1‑301 (MQ=255)`  
       `tgatgaCTTGCCAGACGACTACGAGAAGGTTCGCGGGAGAGTCCCATTCTAATACGACTCACTAAAGGAGACACACCATGTTCAAACTGATTAAGAAGTTAGGCCAACTGCTGGTTCGTATGGACAACGTGGAAGCCAAGCGACTGAACGATGAGGCTCGTAAAGAGGCCACACAGTCACGCGCTCTGGCGATTCGCTCCAACGAACTGGCTGACAGTGCATCCACTAAAGTTACCGAGGCTGCCCGTGTGGCAAACCAAGCTCAACAGCTTTCCAAATTCTTTGAGTAATCAAACAGGAG                                                                                                                                                                                                                                                                                                 <  1:157585‑M1/301‑1 (MQ=255)`  
            `aCTTTCCAGACGACTACGAGAAGTTTCGCTGGAGAGTCCCATTCTAATACGACTCACTAAAGGAGACACACCATGTTCAAACTGATTAAGAAGTTAGGCCAACTGCTGGTTCGTATGTACAACGTGGAAGCAAAGCGACTGAACGATGAGGCTCGTAAAGAGGCCACACAGTCACGCGCTCTGGCGATTCGCTCCAACGAACTGGCTGACAGTGCATCCACTAAAGTTACCGAGGCTGCCCGTGTGGCAAACCAAGCTCAACAGCTTTCCAAATTCTTTGAGTAATCAAACAGGAGAAAtc                                                                                                                                                                                                                                                                                            <  1:677448‑M1/301‑3 (MQ=255)`  
            `aCTTTCCAGACGACTACGAGAAGTTTCGCTGGAGAGTCCCATTCTAATACGAATCACTAAAGGAGACACACCATGTTCAAACTGATTAAGAAGTTAGGCCAACTGCTGGTTCGTATGTACAACGTGGAAGCCAAGCGACTGAACGATGAGGCTCGTAAAGAGGCCACACAGTCACGCGCTCTGGCGATTCGCTCCAACGAACTGGCTGACAGTGCATCCACTAAAGTTACCGAGGCTGCCCGTGTGGCAAACCAAGCTCAACAGCTTTCCAAATTCTTTGAGTAATCAAACAGGAGAAAtc                                                                                                                                                                                                                                                                                            <  1:617009‑M1/301‑3 (MQ=255)`  
               `aagagacagactacgagaaGTTTCGCTGGAGAGTCCCATTCTAATACGACTCACTAAAGGAGACACACCATGTTCAAACTGATTAAGAAGTTAGGCCAACTGCTGGTTCGTATGTACAACGTGGAAGCCAAGCGACTGAACGATGAGGCTCGTAAAGAGGCCACACAGTCACGCGCTCTGGCGATTCGCTCCAACGAACTGGCTGACAGTGCATCCACTAAAGTTACCGAGGCTGCCCGTGTGGCAAACCAAGCTCAACAGCTTTCCAAATTCTTTGAGTAATC‑AATAGGAGAAAtcaata                                                                                                                                                                                                                                                                                        <  1:623169‑M1/293‑7 (MQ=255)`  
                 `ccAGACGACTACGAGAAGTTTCGCTGGAGAGTCCCATTCTAATACGACTCACTAAAGGAGACACACCATGTTCAAACTGATTAAGAAGTTAGGCCAACTGCTGGTTCGTATGTACAACGTGGAAGCCAAGCGACTGAACGATGAGGCTCGTAAAGAGGCCACACAGTCACGCGCTCTGGCGATTCGCTCCAACGAACTGGCTGACAGTGCATCCACTAAAGTTACCGAGGCTGCCCGTGTGGCAAACCAAGCTCAACAGCTTTCCAAATTCTTTGAGTAATCAAACAGGAGAAAtcaataa                                                                                                                                                                                                                                                                                       >  1:809391‑M1/1‑294 (MQ=255)`  
                      `cgacTACGAGAAGTTTCGCTGGAGAGGCCCAGTCTAATACGACACAATAAAGGAGACACACCATGTTCAAACTGAATAAGAAGTTAGGCCAACTGCTGGTTCGTATGTACAGCGTGGAAGCCAAGCGACTGAACGATGAGGCTCGTAAAGAGGTCACACAGTCACGCGCTCTGGCGATTCGCTCCAACGAACTGGCTGACAGTGCATCCACTAAAGTTACCGAGGCTGCCCGTGTGGCAAACCAAGCTCAACAGCTTTCCAAATTCTTTGAGTAATCAAACAGGAGAAAtcaatatgatcg                                                                                                                                                                                                                                                                                  <  1:319480‑M1/301‑13 (MQ=255)`  
                       `gacTACGAGAAGTTTCGCTGGAGAGTCCCATTCTAATACGACTCACTAAAGGAGACACACCATGTTCAAACTGATTAAGAAGTTAGGCCAACTGCTGGTTCGTATGTACAACGTGGAAGCCAAGCGACTGAACGATGAGGCTCGTAAAGAGGCCACACAGTCACGCGCTCTGGCGATTCGCTCCAACGAACTGGCTGACAGTGCATCCACTAAAGTTACCGAGGCTGCCCGTGTGGCAAACCAAGCTCAACAGCTTTCCAAATTCTTTGAGTAATCAAACAGGAGAAAtcaatatgatagt                                                                                                                                                                                                                                                                                 >  1:259606‑M1/1‑288 (MQ=255)`  
                                     `tCGCTGGAGAGTCCCATTCTAATACGACTCACTAAAGGAGACACACCATGTTCAAACTGATTAAGAAGTTAGGCCAACTGCTGGTTCGTATGTACAACGTGGAAGCCAAGCGACTGAACGATGAGGCTCGTAAAGAGGCCACACAGTCACGCGCTCTGGCGATTCGCTCCAACGAACTGGCTGACAGTGCATCCACTAAAGTTACCGAGGCTGCCCGTGTGGCCAACCAAGCTCCACAGCTTTCCCAATTCTTTGAGTAATCAAACAGGCGAAAtcaatatgatcgtttctgccatcgaac                                                                                                                                                                                                                                                                   >  1:721044‑M1/1‑274 (MQ=255)`  
                                      `gtgtataagagacagttctaatacgactcactAAAGGAGACACACCATGTTCAAACTGATTAAGAAGTTAGGCCAACTGCTGGTTCGTATGTACAACGTGGAAGCCAAGCGACTGAACGATGAGGCTCGTAAAGAGGCCACACAGTCACGCGCTCTGGCGATTCGCTCCAACGAACTGGCTGACAGTGCATCCACTAAAGTTACCGAGGCTGCCCGTGTGGCAAACCAAGCTCAACAGCTTTCCAAATTCTTTGAGTAATCAAACAGGAGAAAtcaatatgatcgtttctgacatcgaagc                                                                                                                                                                                                                                                                  <  1:527817‑M1/286‑29 (MQ=255)`  
                                      `cGCTGGAGAGTCCCATTCTAATACGACTCACTAAAGGAGACACACCATGTTCAAACTGATTAAGAAGTTAGGCCAACTGCTGGTTCGTATGTACAACGTGGAAGCCAAGCGACTGAACGATGAGGCTCGTAAAGAGGCCACACAGTCACGCGCTCTGGCGATTCGCTCCAACGAACTGGCTGACAGTGCATCCACTAAAGTTACCGAGGCTGCCCGTGTGGCAAACCAAGCTCAACAGCTTTCCAAATTCTTTGAGTAATCAAACAGGAGAAAtcaatattatcgtttctgacatcgaagc                                                                                                                                                                                                                                                                  <  1:765083‑M1/301‑29 (MQ=255)`  
                                               `gTCCCATTCTAATACGACTCACTAAAGGAGACACACCATGTTCAAACTGATTAAGAAGTTAGGCCAACTGCTGGTTCGTATGTACAACGTGGAAGCCAATCGACTGAACGATGAGGCTCGTAAAGAGGCCACACAGTCACGCGCTCTGGCGATTCGCTCCAACGAACTGGCTGACAGTGCCTCCACTACAGTTACCGAGGCTGCCCGTGTGGCAAACCAAGCTCAACAGCTTTCCAAATTCTTTGAGTAATCAAACAGGAGAAAccattatgatcgtttctgacatcgaagctaacgccct                                                                                                                                                                                                                                                         >  1:670184‑M1/1‑264 (MQ=255)`  
                                               `gTCCCATTCTAATACGACTCACTAAAGGAGACACACCATGTTCAAACTGATTAAGAAGTTAGGCCAACTGCTGGTTCGTATGTACAACGTGGAAGCCAAGCGACTGAACGATGAGGCTCGTAAAGAGGCCACACAGTCACGCGCTCTGGCGATTCGCTCCAACGAACTGGCTGACAGTGCATCCACTAAAGTTACCGAGGCTGCCCGTGTGGCAAACCAAGCTCAACAGCTTTCCAAATTCTTTGAGTAATCAAACAGGAGAAAtcaatatgatcgtttctgacatcgaagctaacgccct                                                                                                                                                                                                                                                         >  1:71359‑M1/1‑264 (MQ=255)`  
                                               `gTCCCATTCTAATACGACTCACTAAAGGAGACACACCATGTTCAAACTGATTAAGAAGTTAGGCCAACTGCTGGTTCGTATGTACAACGTGGAAGCCAAGCGACTGAACGATGAGGCTCGTAAAGAGGCCACACAGTCACGCGCTCTGGCGATTCGCTCCAACGAACTGGCTGACAGTGCATCCACTAAAGTTACCGAGGCTGCCCGTGTGGCAAACCAAGCTCAACAGCTTTCCAAATTCTTTGAGTAATCAAACAGGAGAAAtcaatatgatcgtttctgacatcgaagctaacaccct                                                                                                                                                                                                                                                         >  1:267270‑M1/1‑264 (MQ=255)`  
                                                     `ttCTAATACGACTCACTAAAGGAGACACACCATGTTCAAACTGATTAAGAAGTTAGGCCAACTGCTGGTTCGTATGTACAACGTGGAAGCCAAGCGACTGAACGATGAGGCTCGTAAAGAGGCCACACAGTCACGCGCTCTGGCGATTCGCTCCAACGAACTGGCTGACAGTGCATCCACTACAGTTACCGAGGCTGCCCGTGTGGCAACCCAAGCTCAACAGCTTTCCACATTCTTTGAGTAATCAAACAGGAGAAAtcaatatgatcgtttctgacatcgacgctaacgccctcttaga                                                                                                                                                                                                                                                   >  1:164214‑M1/1‑258 (MQ=255)`  
                                                     `ttCTAATACGACTCACTAAAGGAGACACACCATGTTCAAACTGATTAAGAAGTTAGGCCAACTGCTGGTTCGTATGTACAACGTGGAAGCCAAGCGACTGAACGATGAGGCTCGTAAAGAGGCCACACAGTCACGCGCTCTGGCGATTCGCTCCAACGAACTGGCTGACAGTGCATCCACTAAAGTTACCGAGGCTGCCCGTGTGGCAAACCAAGCTCAACAGCTTTCCAAATTCTTTGAGTAATCAAACAGGAGAAAtcaatatgatcgtttctgacatcgaagctaacgccctcttaga                                                                                                                                                                                                                                                   >  1:346554‑M1/1‑258 (MQ=255)`  
                                                     `ttCTAATACGACTCACTAAAGGAGACACACCATGTTCAAACTGATTAAGAAGTTAGGCCAACTGCTGGTTCGTATGTACAACGTGGAAGCCAAGCGACTGAACGATGAGGCTCGTAAAGAGGCCACACAGTCACGCGCTCTGGCGATTCGCTCCAACGAACTGGCTGACAGTGCATCCACTAAAGTTACCGAGGCTGCCCGTGTGGCAAACCAAGCTCAACAGCTTTCCAAATTCTTTGAGTAATCAAACAGGAGAAAtcaatatgatcgtttctgacatcgaagctaacgccctcttaga                                                                                                                                                                                                                                                   >  1:83610‑M1/1‑258 (MQ=255)`  
                                                     `ttCTAATACGACTCACTAAAGGAGACACACCATGTTCAAACTGATTAAGAAGTTAGGCAAACTGCTGGTTCGTATGTACAACGTGGAAGCCAAGCGACTGAACGATGAGGCTCGTAAAGAGGCTACACAGTCACGCGCTCTGGCGATTCGCTCCAACGAACTGGCTGACAGTGCATCCACTAAAGTTACCGAGGCTGCCCGTGTGGCAAACCAAGCTCAACAGCTTTCCAAATTCTTTGAGTAATCAAACAGGAGAAAtcaatatgatcgtttctgacatcgaagctaacgccctcttaga                                                                                                                                                                                                                                                   >  1:173350‑M1/1‑258 (MQ=255)`  
                                                     `ttCTAATACGACTAACTAAAGGAGACACACCATGTTCAAACTGATTAAGAAG‑TAGGCCAACTGCTGGTTCGTATGTACAACGTGGAAGCCAAGCGACTGAACGATGAGGCTCGTAAAGAGGCCACACAGTCACGCGCTCTGGCGATTCTCTCCAACGAACTGGCTGACAGTGCATCCACTAAAGTTACCGAGGCTGCCCGTGTGGCAAACCAAGCTCAACAGCTTTCCAAATTCTTTGAGTAATCAAACAGGAGAAAtcaatatgatcgtttctgacatcgaagctaacgccctcttagag                                                                                                                                                                                                                                                  <  1:151101‑M1/301‑45 (MQ=255)`  
                                                      `tgtgtataacagacagaaaggagacacaccatgttCAAACTGATTAAGAAGTTAGGCCAACTGCTGGTTCGTATGTACAACGTGGAAGCCAAGCGACTGAACGATGAGGCTCGTAAAGAGGCCACACAGTCACGCGATCTGGCGATTCGCTCCAACGAACTGGCTGACAGTGCATCCACTAAAGTTACCGAGGCTGCCCGTGTGGCAAACCAAGCTCAACAGCTTTCCAAATTCTTTGAGTAATCAAACAGGAGAAAtcaatatgatcgtttctgacatcgaagctaacgccctcttagag                                                                                                                                                                                                                                                  <  1:685922‑M1/285‑45 (MQ=255)`  
                                                      `tCTAATACGACTCACTAAAGGAGACACACCATGTTCAAACTGATTAAGAAGTTAGGCCAACTGCTGGTTCGTATGTACAACGTGGAAGCCAAGCGACTGAACGATGAGGCTCGTAAAGAGGCCACACAGTCACGCGCTCTGGCGATTCGCTCCAACGAACTGGCTGACAGTGCGTCCACTAAAGTTACCGAGGCTGCCCGTGTGGCAAACCAAGCTCAACAGCTTTCCAAATTCTTTGAGTAATCAAACAGGAGAAAtcaatatgatcgtttctgacatcgaagctaacgccctcttagag                                                                                                                                                                                                                                                  <  1:705913‑M1/301‑45 (MQ=255)`  
                                                      `tCTAATACGACTCACTAAAGGAGACACACCATGTTCAAACTGATTAAGAAGTTAGGCCAACTGCTGGTTCGTATGTACAACGTGGAAGCCAAGCGACTGAACGATGAGGCTCGTAAAGAGGCCACACAGTCACGCGCTCTGGCGATTCGCTCCAACGAACTGGCTGACAGTGCATCCACTAAAGTTACCGAGGCTGCCCGTGTGGCAAACCAAGCTCAACAGCTTTCCAAATTCTTTGAGTAATCAAACAGGAGAAAccaatatgatcgtttctgacatcgaagctaacgccctcttagag                                                                                                                                                                                                                                                  <  1:181156‑M1/301‑45 (MQ=255)`  
                                                      `tCTAATACGACCCACTAAAGGAGACACACCATGTTCAAACTGATTAAGAAGTTAGGCCAACTGCTGGTTCGTATGTACAACGTGGAAGCCAAGCGACTGAACGATGAGGCTCGTAAAGAGGCCACACAGTCACGCGCTCTGGCGATTCGCTCCAACGAACTGGCTGACAGTGCATCCACTAAAGTTACCGAGGCTGCCCGTGTGGCAAACCAAGCTCAACAGCTTTCCAAATTCTTTGAGTAATCAAACAGGAGAAAtcaatatgatcgtttctgacatcgaagctaacgccctcttagag                                                                                                                                                                                                                                                  <  1:603843‑M1/301‑45 (MQ=255)`  
                                                      `agatgtggataagggacagggagacacaccatgttcaaacTGATTAAGAAGTTAGGCCAACTGCTGGTTCGTATGTACAACGAGGAAGCCAAGCGACTGAACGATGAGGCTCGTAAAGAGGCCACACAGTCACGCACTCTGGCGATTCGCTCCAGCGAACTGGCTGACAGTGCATCCACTAAAGTTACCGAGGCTGCCCGTGTGGCAAACCAAGCTCAACAGCTTTCCAAATTCTTTGAGTAATCAAACAGGAGAAAacaatatgatcgtttctgacatcgaagctaacgctctcttagag                                                                                                                                                                                                                                                  <  1:142599‑M1/282‑45 (MQ=255)`  
                                                      `aagagacagactcactaAAGGAGACACACCATGTTCAAACTGATTAAGAAGTTAGGCCAACTGCTGGTTCGTATGTACAACGTGGAAGCCAAGCGACTGAACGATGAGGCTCGTAAAGAGGCCACACAGTCACGCGCTCTGGCGATTCGCTCCAACGAACTGGCTGACAGTGCATCCACTAAAGTTACCGAGGCTGCCCGTGTGGCAAACCAAGCTCAACAGCTTTCCAAATTCTTTGAGTAATCAAACAGGAGAAAtcaatatgatcgtttctgacatcgaagctaacgccctcttagag                                                                                                                                                                                                                                                  <  1:28942‑M1/293‑45 (MQ=255)`  
                                                                  `cACTAAAGGAGACACACCATGTTCAAACTGATTAAGAAGTTAGGCCAACTGCTGGTTTGTATGTACAACGTGGAAGCCAAGCGACTGAACGATGAGGCTCGTAAAGAGGCCACACAGTCACGCGCTCTGGCGATTCGCTCCAACGAACTGGCTGACAGTGCATCCACTAAAGTTATCGAGGTTGCCCGTGTGGCAAACCAAGCTCAACAGCTTTCCAAATTCTTTGAGTAATCAAACAGTAGAAAtcaatctgaccgtttctgacatcacagctaacgccctcttagcgactgccttcaat                                                                                                                                                                                                                                      >  1:498672‑M1/1‑245 (MQ=255)`  
                                                                  `cACTAAAGGAGACACACCATGTTCAAACTGATTAAGAAGTTAGGCCAACTGCTGGTTCGTATGTACAACGTGGAAGCCAAGCGACTGAACGATGAGGCTCGTAAAGAGGCCACACAGTCACGCGCTCTGGCTATTCGCTCCAACGAACTGGCTGACAGTGCATCCACTAAAGTTACCGAGGCTGCCCGTGTGGCAAACCAAGCTCAACAGCTTTCCAAATTCTTTGAGTAATCAAACAGGAGAAAtcaatatgatcgtttctgacatcgaagctaacgccctcttagagagcgtcactaag                                                                                                                                                                                                                                      >  1:223048‑M1/1‑245 (MQ=255)`  
                                                                  `cACTAAAGGAGACACACCATGTTCAAACTGATTAAGAAGTTAGGCCAACTGCTGGTTCGTATGTACAACGTGGAAGCCAAGCGACTGAACGATGAGGCTCGTAAAGAGGCCACACAGTCACGCGCTCTGGCGATTCGCTCCAACGAACTGGCTGACAGTGCATCCACTAAAGTTACCGAGGCTGCCCGTGTGGCAAACCAAGCTCAACAGCTTTCCAAATTCTTTGAGTAATCAAATAGGAGAAAtcaatatgatcgtttctgacatcgaagctaacgccctcttagagagcgtcactaag                                                                                                                                                                                                                                      >  1:59483‑M1/1‑245 (MQ=255)`  
                                                                  `cACTAAAGGAGACACACCATGTTCAAACTGATTAAGAAGTTAGGCCAACTGCTGGTTCGTATGTACAACGTGGAAGCCAAGCGACTGAACGATGAGGCTCGTAAAGAGGCCACACAGTCACGCGCTCTGGCGATTCGCTCCAACGAACTGGCTGACAGTGCATCCACTAAAGTTACCGAGGCTGCCCGTGTGGCAAACCAAGCTCAACAGCTTTCCAAATTCTTTGAGTAATCAAACAGGAGAAAtcaatatgatcgtttctgacatcgaagctaacgcactcttagagagcgtcactaag                                                                                                                                                                                                                                      >  1:69923‑M1/1‑245 (MQ=255)`  
                                                                     `aaaagGAGACACACCAAGTTCAAACTGATTAAGAAGTTAGGCCAACTGCTGGTTCGTATGTACAACGTGGAAGCCAAGCGACTGAACGATGAGGCTCGTAAAGAGGCCACACAGTCACGCGCTTTGGCGATTCGCTCCAACGAACTGGCTGACAGTGCATCCACTAAAGTTACCGAGGCTGCCCGTGTGGCAAACCAAGCTCAACAGCTTTCCAAATTCTTTGAGTAATCAAACAGGAGAAAtcaatatgatcgtttctgacatcgaagctaacgccctcttagagagcgtaactaagttc                                                                                                                                                                                                                                   <  1:611319‑M1/300‑60 (MQ=255)`  
                                                                      `aaaGGAGACACACCATGTTCAAACTGATTAAGAAGTTAGGCCAACTGCTGGTTCGTATGTACAACGTGGAAGCCAAGCGACTGAACGATGAGGCTCGTAAAGAGGCCACACAGTCACGCGCTCTGGCGATTCGCTCCAACGAACTGGCTGACAGTGCATCCACTAAAGTTACCGAGGCTGCCCGTGTGGCAAACCAAGCTCAACAGCTTTCCAAATTCTTTGAGTAATCAAACAGGAGAAAtcaatatgatcgtttctgacatcgaagctaacgccctcttagagcgcgtcactaagttcc                                                                                                                                                                                                                                  >  1:588424‑M1/1‑241 (MQ=255)`  
                                                                       `ggggggctcggaaatgtgtataagagacaggaatttaggccaaatgatggttcgtatgtaaaacGGGGAAGACAAGCGACTGAACGAGGAGGCTCGTAAAGAGGCCACACAGTCACGCGATCTGGCGATTCGCTCCAACGAACTGGCTGACAGTGCATCCACTAAAGTTACCGAGGCTGCCCGGGTGGCAAACCAAGCTCAACAGCTTTCCAAATTCTTTGAGTAATC‑AATAGGAGAAAtcaatatgatcgtttctgacatcgaagctaacgccctcttagagagcgtcactaagttccac                                                                                                                                                                                                                                <  1:483221‑M1/271‑63 (MQ=255)`  
                                                                         `ggAGACACACCATGTTCAAACTGATTAAGAAGTTAGGCCAACTGCTGGTTCGTATGTGCAACGTGGAAGCCAAGCGACTGAACGATGAGGCTCGTAAAGAGGCCACACAGTCACGCGCTCTGGCGATTCGCTCCAACGAACTGGCTGACAGTGCATCCACTAAAGTTACCGAGGCTGCCCGTGTGGCAAACCAAGCTCAACAGCTTTCCAAATTCTTTGAGTAATCAAACAGGAGAAAtcaatatgatcgtttctgacatcgaagctaacgccctcttagagctgtctcttatacacatct                                                                                                                                                                                                                               >  1:631650‑M1/1‑238 (MQ=255)`  
                                                                         `ggAGACACACCATGTTCAAACTGATTAAGAAGTTAGGCCAACTGCTGGTTCGTATGTACAACGTGGAAGCCAAGCGACTGAACGATGAGGCTCGTAAAGAGGCCACACAGTCACGCGCTCTGGCGATTCGCTCCAACGAACTGGCTGACAGTGCATCCACTAAAGTTACCGAGGCTGCCCGTGTGGCAAACCAAGCTCAACAGCTTTCCAAATTCTTTGAGTAATCAAACAGGAGAAAccattatgatcgtttctgacatcgaagctaacgccctcttagagctgtctcttatacacatct                                                                                                                                                                                                                               >  1:422859‑M1/1‑238 (MQ=255)`  
                                                                          `gagaCACACCATGTTCAAACTGATTAAGAAGTTAGGCCAACTGCTGGTTCGTATGTACAACGTGGAAGCCAAGCGACTGAACGATGAGGCTCGTAAAGAGGCCACACAGTCACGCGCTCTGGCGATTCGCTCCAACGAACTGGCTGACAGTGCATCCACTAAAGTTACCGAGGCTGCCCGTGTGGCAAACCAAGCTCAACAGCTTTCCAAATTCTTTGAGTAATCAAACAGGAGAAAtcaatatgatcgtttctgacatcgaagctaacgccctcttagagctgtctcttatacacatctc                                                                                                                                                                                                                              >  1:372443‑M1/1‑237 (MQ=255)`  
                                                                          `gagaCACACCATGTTCAAACTGATTAAGAAGTTAGGCCAACTGCTGGTTCGTATGTACAACGTGGAAGCCAAGCGACTGAACGATGAGGCTCGTAAAGAGGCCACACAGTCACGCACTCTGGCGATTCGCTCCAACGAACTGGCTGACAGTGCATCCACTAAAGTTACCGAGGCTGCCCGTGTGGCAAACCAAGCTCAACAGCTTTCCAAATTCTTTGAGTAATCAAACAGGAGAAAtcaatatgatcgtttctgacatcgaagctaacgccctcttagagagcgtcactaagttcctgtc                                                                                                                                                                                                                              >  1:490374‑M1/1‑237 (MQ=255)`  
                                                                          `ataagagacagtgttcaaactgaTTAAGAAGTTAGGCCAACTGCTGGTTCGTATGTACAACGTGGAAGCCAAGCGACTGAACGATGAGGCTCGTAAAGAGGCCACACAGTCACGCGCTCTGGCGATTCGCTCCAACGAACTGGCTGACAGTGCATCCACTAAAGTTACCGAGGCTGCCCGTGTGGCAAACCAAGCTCAACAGCTTTCCAAATTCTTTGAGTAATCAAACAGGAGAAAtcaatatgatcgtttctgacatcgaagctaacgccctcttagagagcgtcactaagttccactg                                                                                                                                                                                                                              <  1:51331‑M1/290‑65 (MQ=255)`  
                                                                                `cacCATGTTCAAACTGATTAAGAAGTTAGGCCAACTGCTGGTTCGTATGTACAACGTGGAAGCCAAGCGACTGAACGATGAGGCTCGTAAAGAGGCCACACAGTCACGCGCTCTGGCGATTCGCTCCAACGAACTGGCTGACAGTGCATCCACTAAAGTTACCGAGGCTGCCCGTGTGGCAAACCAAGCCCAACAGCTTTCCAAATTCTTTGAGTAATCAAACAGGAGAAAtcaatatgatcgtttctgacatcgaagctaacgccctcttagagagcgtcactaagttccactgcggggg                                                                                                                                                                                                                        >  1:332883‑M1/1‑231 (MQ=255)`  
                                                                                    `gagatgtgtataagagacaggttaggccaactgctggttcgTATGTACAACGTGGAAGCCAAGCGACTGAACGATGAGGCTCGTAAAGAGGCCACACAGTCACGCGCTCTGGCGATTCGCTCCAACGAACTTGCTGACAGTGCATCCACTAAAGTTACCGAGGCTGCCCGTGTGGCAAACCAAGCTCAACAGCTTTCCAAATTCTTTGAGTAATCAAACAGGAGAAAtcaatatgatcgtttctgacatcgaagctaacgccctcttagagagcgtcactaagttccactgcggggttatc                                                                                                                                                                                                                    <  1:557262‑M1/281‑75 (MQ=255)`  
                                                                                     `tGTTCAAACTGATTAAGAAGTTAGGCCAACTGCTGGTTCGTATGTACAACGTGGAAGCCAAGCGACTGAACGATGAGGCTCGTAAAGAGGCCACACAGTCACGCGCTCTGGCGATTCGCTCCAACGAACTGGCTGACAGTGCATCCACTAAAGTTACCGAGGCTGCCCGTGTGGCAAACCAAGCTCAACAGCTTTCCAAATTCTTTGAGTAATCAAACAGGAGAAAtcaatatgatcgtttctgacatcgaagctaacgccctcttagagagcgtcactaagttccactgcgggtttatct                                                                                                                                                                                                                   >  1:507024‑M1/1‑226 (MQ=255)`  
                                                                                       `ttCAAACTGATTAAGAAGTTAGGCCAACTGCTGTTTCGTATGTACAACGTGGAAGCCAAGCGACTGAACGATGAGGCTCGTAAAGAGGCCACACAGTCACGCGCTCTGGCGATTCGCTCCAACGAACTGGCTGACAGTGCATCCACTAAAGTTACCGAGGCTGCCCGTGTGGCAAACCAAGCTCAACAGCTTTCCAAATTCTTTGAGTAATCAAACAGGAGAAAtcaatatgatcgtttctgacatcgaagctaacgccctcttagagagcgtcactaagttccactgcggggttatctac                                                                                                                                                                                                                 <  1:208080‑M1/301‑78 (MQ=255)`  
                                                                                       `ttCAAACTGATTAAGAAGTTAGGCCAACTGCTGGTTCGTATGTACAACGTGGAAGCCAAGCGACTGAACGATGAGGCTAGTAAAGAGGCCACACAGTCACGCGCTCTGGCGATTCGCTCCAACGAACTGGCTGACAGTGCATCCACTAAAGTTACCGAGGCTGCCCGTGTGGCAAACCAAGCTCAACAGCTTTCCAAATTCTTTGAGTAATCAAACAGGAGAAAtcaatatgatcgtttctgatatcgaagctaacgccctcttagagagcgtcactaagttccactgcggggttatctac                                                                                                                                                                                                                 <  1:274669‑M1/301‑78 (MQ=255)`  
                                                                                          `gacaggattaaGAAGTTAGGCCAACTGCTGGTTCGTAGGTACAACGTGGAAGCCAAGCGACTGAACGATGAGGCTCGTAAAGAGGCCACACAGTCACGCGCTCTGGCGATTCGCTCCAACGAACTGGCTGACAGTGCATCCACTAAAGTTACCGAGGCTGCCCGTGTGGCAAACCAAGCTCAACAGCTTTCCAAATTCTTTGAGTAATCAAACAGGAGAAAtcaatatgatcgtttctgacatcgaagctaacgccctcttagagagcgtcactaagttccactgcggggttatctacgac                                                                                                                                                                                                              <  1:822314‑M1/296‑81 (MQ=255)`  
                                                                                               `gATTAAGAAGTTAGGCCAACTTCTGGTTCGTATGTACAACGTGGAAGCCAAGCGACTGAACGATGAGGCTCGTAAAGAGGCCACACAGTCACGCTCTCTGGCGATTCGCTCCAACGAACTGGCTGACAGTGCATCCACTAAAGTTACCGAGGCTGCCCGTGTGGCAAACCAAGCTCAACAGCTTTCCAAATTCTTTGAGTAATCAAACAGGAGAAAtcaatatgatcgtttctgacatcgaagctaacgccctcttagagagcgtcactaagttccactgcggggttatctacgactactc                                                                                                                                                                                                         <  1:430306‑M1/301‑86 (MQ=255)`  
                                                                                               `gATTAAGAAGTTAGGCCAACTGCTGGTTCGTATGTACAACGTGGAAGCCAAGCGACTGAACGATGAGGCTCGTAAATAGGCCACACAGTCACGCGCTCTGGCGATTCGCTCCAACGAACTGGCGGACAGTGCATCCACTAAAGTTACCGAGGCTGCCCGGGTGGCAAACCAAGCTCAACAGCTTTCACAATTCTTTGAGTAATCAAACAGGAGAACtcaatatgatcgcttatgacatcgaagctaacgccctcttagagagcgtccataacttccactacggggttatctccgactactc                                                                                                                                                                                                         >  1:476861‑M1/1‑216 (MQ=255)`  
                                                                                               `gATTAAGAAGTTAGGCCAACTGCTGGTTCGTATGTACAACGTGGAAGCCAAGCGACTGAACGATGAGGCTCGTAAAGAGGCCACACAGTCACGCGCTCTGGCGATTCGCTCCAACGAACTGGCTGACAGTGCATCCACTAAAGTTACCGAGGCTGCCCGTGTGGCAAACCAAGCTCAACAGCTTTCCAAATTCTTTGAGTAATCAAACAGGAGAAAtcaatatgatcgtttctgacatcgaagctaacgccctcttagagagcgtcactaagttccactgcggggttatctacgacctgtc                                                                                                                                                                                                         >  1:120945‑M1/1‑216 (MQ=255)`  
                                                                                                `gtataagagacaggccaactgctGGTTCGTATGTACAACGTGGAAGCCAAGCGACTGAACGATGAGGCTCGGAAAGAGGCCACACAGTCACGCGCTCTGGCGATTCGCTCCAACGAACTGGCTGACAGTGCATCCACTAAAGTTACCGAGGCTGCCCGTGTGACAAACCAAGCTCAACAGCTTTCCAAATTCTTTGAGTAATCAAACAGGAGAAAtcaatatgatcgtttctgacatcgaagctaacgccctcttagagagcgtcactaagttccactgcggggttatctacgactactcc                                                                                                                                                                                                        <  1:571497‑M1/290‑87 (MQ=255)`  
                                                                                                `aTTAAGAAGTTAGGCCAACTGCTGGTTCGTATGTACAACGTGGAAGCCAAGCGACTGAACGATGAGGCTCGTAAAGAGGCCACACAGTCACGCGCTCTGGCGATTCGCTCCAACGAACTGGTTGACAGTGCATCCACTAAAGTTACCGAGGCTGCCCGTGTGGCAAACCAAGCTCAACAGCTTTCCAAATTCTTTGAGTAATCAAACAGGAGAAAccattatgatcgtttctgacatcgaagctaacgccctcttagagagcgtcactaagttccactgcggggttatctacgactactcc                                                                                                                                                                                                        >  1:243269‑M1/1‑215 (MQ=255)`  
                                                                                                          `agggccAACTGCTGGTTCGTATGTACAACGTGGAAGCCAAGCGACTGAACGATGAGGCTCGTAAAGAGGCCACACAGTCACGCGCTCTGGCGATTCGCTCCAACGAACTGGCTGACAGTGCATCCACTAAAGTTACCGAGGCTGCCCGTGTGGCAAACCAAGCTCAACAGCTTTCCAAATTCTTTGAGTAATCAAACAGGAGAAAtcaatatgatcgtttctgacatcgaagctaacgccctcttagagagcgtcactaagttccactgcggggttatctacgactactccaccgctgagt                                                                                                                                                                                              <  1:252058‑M1/299‑97 (MQ=255)`  
                                                                                                                     `tgtgtataagagacagacgtggaagccaagcgaCTGAACGATGAGGCTCGTAAAGAGGCCACACAGTCACGCGCTCTGGCGATTCGCTCCAACGAACTGGCTGACAGTGCATCCACTAAAGTTACCGAGGCTGCCCGTGTGGCAAACCAAGCTCAACAGCTTTCCAAATTCTTTGAGTAATCAAACAGGAGAAAccattatgatcgtttctgacatcgaagctaacgccctcttagagagcgtcactaagttccactgcggggttatctacgactactccaccgctgagtacgtaagctac                                                                                                                                                                                   <  1:388364‑M1/285‑108 (MQ=255)`  
                                                                                                                        `gTTCGTATGTACAACGTGGAAGCCAAGCGACTGAACGATGAGGCTCGTAAAGAGGCCACACAGTCACGCGCTCTGGCGATTCGCTCCAACGAACTGGCTGACAGTGCATCCACTAAAGTTACCGAGGCTGCCCGTGTGGCAAACCAAGCTCAACAGCTTTCCAAATTCTTTGAGTAATCAAACAGGAGAAAccattatgatcgtttctgacatcgaagctaacgccctcttagagagcgtcactaagttccactgcggggttatctacgactactccaccgctgagtacgtaagctaccgt                                                                                                                                                                                >  1:325211‑M1/1‑191 (MQ=255)`  
                                                                                                                           `cGTATGTACAACGTGGAAGCCAAGCGACTGAACGATGAGGCTCGTAAAGAGGCCACACAGTCACGCGCTCTGGCGATTCGCTCCAACGAACTGGCTGACAGTGCATCCACTAAAGTTACCGAGGCTGCCCGTGTGGCAAACCAAGCTCAACAGCTTTCCAAATTCTTTGAGTAATCAAACAGGAGAAAtcaatatgatcgtttctgacatcgaagctaacgccctcttagagagcgtcactaagttccactgcggggttatctacgactactccaccgctgagtacgtaagctgtctcttt                                                                                                                                                                             >  1:20031‑M1/1‑188 (MQ=255)`  
                                                                                                                           `cGTATGTACAACGTGGAAGCCAAGCGACTGAACGATGAGGCTCGTAAAGAGGCCACACAGTCACGCGCTCTGGCGATTCGCTCCAACGAACTGGCTGACAGTGCATCCACTAAAGTTACCGAGGCTGCCCGTGTGGCAAACCAAGCTCAACAGCTTTCCAAATTCTTTGAGTAATCAAACAGGAGAAAtcaatatgatcgtttctgacatcgaagctaacgccctcttagagagcgtcactaagttccactgcggggttatctacgactactccaccgctgagtacgtaagctaccgtccg                                                                                                                                                                             >  1:551093‑M1/1‑188 (MQ=255)`  
                                                                                                                           `cGTATGTACAACGTGGAAGCCAAGCGACTGAACGATGAGGCTCGTAAAGAGGCCACACAGTCACGCGCTCTGGCGATTCGCTCCAACGAACTGGCTGACAGTGCATCCACTAAAGTTACCGAGGCTGCCCGTGTGGCAAACCAAGCTCAACAGCTTTCCAAATTCTTTGAGTAATCAAACAGGAGAAAccattatgatcgtttctgacatcgaagctaacgccctcttagagagcgtcactaagttccactgcggggttatctacgactactccaccgctgagtacgtaagctaacgtccg                                                                                                                                                                             >  1:305429‑M1/1‑188 (MQ=255)`  
                                                                                                                                `gTACAACGTGGAAGCCAAGCGACTGAACGATGAGGCTCGTAAAGAGGCCACACAGTCACGCGCTCTGGCGATTCGCTCCAACGAACTGGCTGACAGTGCATCCACTAAAGTTACCGAGGCTGCCCGTGTGGCAAACCAAGCTCAACAGCTTTCCAAATTCTTTGAGTAATCAAACAGGAGAAAtcaatatgatcgtttctgacatcgaagctaacgccctcttagagagcgtcactaagttccactgcggggttatctacgactactccaccgctgagtacgtaagctaccttccgagtga                                                                                                                                                                        >  1:692544‑M1/1‑183 (MQ=255)`  
                                                                                                                                   `agacgTGGAAGCCAAGCGACTGAACGATGAGGCTCGTAAAGAGGCCACACAGTCACGCGCGCTGGCGATTCGCTCCAACTAACGGGCTGACAGTGCATCCACTAAAGTTACCGAGGCTGCCCGTGTGGCAAACCAAGCTCAACAGCTTTCCAAATTCTTTGAGTAATCAAACAGGAGAAAtcaatatgatcgtttctgacatcgaagctaacgccctcttagagagcgtcactaagttccacagcggggttatctacgactactccaccgctgagtacgtaagctaccgtccgagtgactt                                                                                                                                                                     <  1:661722‑M1/299‑122 (MQ=255)`  
                                                                                                                                    `gtgggctcggagatgtgtataagagacagggctcgtaaagaggccacacagtcacgcgctCTGGCGATTCGCTCCAACGAACTGGCTGACAGTGCATCCACTAAAGTTACCGAGGCTGCCCGTGTGGCAAACCAAGCTCAACAGCTTTCCAAATTCTTTGAGTAATCAAACAGGAGAAAtcaatatgatcgtttctgacatcgaagctaacgccctcttagagagcgtcactaagttccactgcggggttatctacgactactccaccgctgagtacgtaagctaccgtccgagtgacttc                                                                                                                                                                    <  1:793161‑M1/272‑123 (MQ=255)`  
                                                                                                                                    `aaCGTGGAAGCCAAGCGACTGAACGATGAGGCTCGTAAAGAGGCCACACAGTCACGCGCTCTGGCGATTCGCTCCAACGAACTGGCTGACAGTGCATCCACTAAAGTTACCGAGGCTGCCCGTGTGGCAAACCAAGCTCAACAGCTTTCCAAATTCTTTGAGTAATCAAACAGGAGAAAtcaatatgatcgtttctgacatcgaagctaacgccctcttagagagcgtcactaagttccactgcggggttatctacgactactccaccgctgagtacgtaagctaccgtccgagtgacttc                                                                                                                                                                    <  1:92227‑M1/301‑123 (MQ=255)`  
                                                                                                                                     `aCGTGGAAGCCAAGCGACTGAACGATGAGGCTCGTAAAGAGGCAACACAGTCACGCGCTCGGGCGATTCGCTCCAACGAACTGGCTGACAGTGCATCCACTAAAGTTACCGAGGCTGCCCGTGTGGCAAACCAAGCTCAACAGCTGTCCAAATTCTTTGAGTAATCAAACAGGTAAAAtcaatatgatcgtttctgacatcgaagctaacgccctcttagagagcgtcactaagttccactgcggggttatctacgactactccaccgctaggtacgtaagcaaccttccgagtgacttcc                                                                                                                                                                   >  1:363385‑M1/1‑178 (MQ=255)`  
                                                                                                                                         `ggAAGCCAAGCGACTGAACGATGAGGCTCGTAAAGAGGCCACACAGTCACGCGCTCTGGCGATTCGCTTCAACGAACTGGCTGACAGTGCATCCACTAAAGTTACCGAGGCTGCCCGTGTGGCAAACCAAGCTCAACAGCTTTCCAAATTCTTTGAGTAATCAAACAGGAGAAAtcaatatgatcgtttctgacatcgaagctaacgccctcttagagagcgtcactaagttccactgcggggttatctacgactactccaccgctgagtacgtaagctaccgtccgagtgacttcggtgc                                                                                                                                                               <  1:800646‑M1/301‑128 (MQ=255)`  
                                                                                                                                          `gAAGCCAAGCGACTGAACGATGAGGCTCGTAAAGAGGCCACACAGTCACGAGCTCTAGCGATTGTCTCCAACGAACTGGCTGACAGTGCATCCACTAAAGTTACCGAGGCTGCCCGTGTGGCAAACCAAGCTCAACAGCTTTCCAAATTCTTTGAGTAATCAAACAGGAGAAAtcaatatgatcgtttctgacatcgaagctaacgccctcttagagagcgtcactaagttccactgcggggttatctacgactactccaccgctgagtacgtaagctaccgtccgagtgacttcggtgcg                                                                                                                                                              <  1:194367‑M1/301‑129 (MQ=255)`  
                                                                                                                                                  `gCGACTGAACGATGAGGCTCGTAAAGAGGCCACACAGTCACGCGCTCTGGCGATTCGCTCCAACGAACTGGCTGACAGTGCATCCACTAAAGTTACCGAGGCTGCCCGTGTGGCAAACCAAGCTCAACAGCTTTCCAAATTCTTTGAGTAATCAAACAGGAGAAAtcaatatgatcgtttctgacatcgaagctaacgccctcttagagagcgtcactaagttccactgcggggttatctacgactactccaccgctgagtacgtaagctaccgtccgagtgacttcggtgcgtatctgga                                                                                                                                                      <  1:469427‑M1/301‑137 (MQ=255)`  
                                                                                                                                                            `gATGAGGCTCGTAAAGAGGCCACACAGTCACGCGCTCTGGCGATTCGCTCCAACGAACTGGCTAACAGTGCATCCACTAAAGTTACCGAGGCTGCCCGTGTGGCAAAACAAGCTCAACAGCTTTCCAAATTCTATGAGTAATCAAACAGGAGAAAtcaatatgatcgtttctgacatcgaagctaacgccctcttagagagcgtcactaagttccactgcggggttatctacgactgcgccaccgctgagtacgtaagctaccgtccgagtgacttcggtgcgtatctggatgcgctggaa                                                                                                                                            <  1:404679‑M1/301‑147 (MQ=255)`  
                                                                                                                                                              `tGAGGCTCGTAAAGAGGCCACACAGTCACGCGCTCTGGCGATTCGCTCCAACGAACTGGCTGACAGTGCATCCACTAAAGTTACCGAGGCTGCCCGTGTGGCAAACCAAGCTCAACAGCTTTCCAAATTCTTTGAGTAATCAAACAGGAGAAAccaatatgatcgtttctgacatcgaagctaacgccctcttagagagcgtcactaagttccactgcggggttatctacgactactccaccgctgagtacgtaagctaccgtccgagtgacttcggtgagtatctggatgcgctggaagc                                                                                                                                          <  1:639063‑M1/301‑149 (MQ=255)`  
                                                                                                                                                                   `cTCGGAAAGAGGCCACACAGTCACGCGCTCTGGCGATTCGCTCCAACGAACTGGCTGACAGTGCATCCACTAAAGTTACCGAGGCTGCCCGTGTGGCAAACCAAGCTCAACAGCTTTCCAAATTCTTTGAGTAATCAAACAGGAGAAAtcaatatgatcgtttctgacatcgaagctaacgccctcttagagagcgtcactaagttccactgcggggttatctacgactactccaccgctgagtacgtaagctaccgtccgagtgacttcggtgcgtatctggatgcgctggaagccgagg                                                                                                                                     <  1:816140‑M1/301‑154 (MQ=255)`  
                                                                                                                                                                                    `caGTCACGCGCTCTGGCGATTCGCTCCAACGAACTGGCTGACAGTGCATCCACTGAAGTTACCGAGGCTGCCCGTGTGGCAAACCAAGCTCAACAGCTTTCCAAATTCTTTGAGTAATCAAACAGGAGAAAtcaatatgatcgtttctgacatcgaagctaacgccctcttagagagcgtcactaagctccactgcggggttatctacgactactccaccgctgagtacgtaagctaccgtccgagtgacttcggtgcgtatctggatgcgctggaagccgaggttgcacgaggcggtctt                                                                                                                    <  1:297306‑M1/301‑171 (MQ=255)`  
                                                                                                                                                                                        `cACGCGCTCTGGCGATTCGCTCCAACGAACTGGCTGACAGTGCATCCACTAAAGTTACCGAGGCTGCCCGTGTGGCAAACCAAGCTCAACAGCTTTCCAAATTCTTTGAGTAATCAAACAGGAGAAAtcaatatgatcgtttctgacatcgaagctaacgccctcttagagagcgtcactaagttccactgcggggttatctacgactactccaccgctgagtacgtaagctaccgtccgagtgacttcggtgcgtatctggatgcgctggaagccgaggttgcacgaggcggtcttattg                                                                                                                <  1:381301‑M1/301‑175 (MQ=255)`  
                                                                                                                                                                                        `cACGCGCTCTGGCGATTCGCTCCAACGAACTGGCTGACAGTGCATCCACTAAAGTTACCGAGGCTGCCCGTGTGGCAAACCAAGCTCAACAGCTTTCCAAATTCTTTGAGTAATCAAACAGGAGAAAtcaatatgatcgtttctgacatcgaagctaacgccctcttagagagcgtcactaagttccactgcggggttatctacgactactccaccgctgagtacgtaagctaccgtccgagtgacttcggtgcgtatctggatgcgctggaagccgaggttgcacgaggcggtcttattg                                                                                                                <  1:312890‑M1/301‑175 (MQ=255)`  
                                                                                                                                                                                        `cACGCGCTCTGGCGATTCGCTCCAACGAACTGGCTGACAGTGCATCCACTAAAGTTACCGAGGCTGCCCGTGTGGCAAACCAAGCTCAACAGCTTTCCAAATTCTTTGAGTAATCAAACAGGAGAAAtcaatatgatcgtttctgacatcgaagctaacgccctcttagagagcgtcactaagttccactgcggggttatctacgactactccaccgctgagtacgtaagctaccgtccgagtgacttcggtgcgtatctggatgcgctggaagccgaggttgcacgaggaggtcttattg                                                                                                                <  1:771357‑M1/301‑175 (MQ=255)`  
                                                                                                                                                                                                         `gtgggctcggagatgtgtataagagacagcacgaaagttaccgaggctgcccgtgtggcAAACCAAGCTCAACAGCTTTCCAAATTCTTTGAGT‑‑‑‑‑‑‑‑‑‑‑‑‑‑AAtcaatatgatcgtttctgacatcgaagctaacgccctcttagagagcgtcactaagttccactgcggggttatctacgactactccaccgctgagtacgtaagctaccgtccgagtgacttcggtgcgtatctggatgcgctggaagccgaggttgcacgaggcggtcttattgtgttccacaacggtcacaagtatgacgttcc                                                                                 <  1:728189‑M1/272‑206 (MQ=255)`  
                                                                                                                                                                                                                    `gagatgtgtataagagacagctaaagtgaccgaggctgcccGTGTGGCAAACCAAGCTAAACAGCTTTCCAAATTCTTTGAGTAATC‑AATAGGAGAAAtcaatatgatcgtttctgacatcgaagctaacgccctcttagagagcgtcactaagttccactgcggggttatctacgactactccaccgctgagtacgtaagctaccgtccgagtgacttcggtgcgtatctggatgcgctggaagccgaggttgcacgaggcggtcttattgtgttccacaacggtcacaagtatgacgtt                                                                                   <  1:414507‑M1/281‑204 (MQ=255)`  
                                                                                                                                                                                                                     `cTGGCTGACAGTGCATCCACTAAAGTTACCGAGGCTGCCCGTGTGGCAAACCAAGCTCAACAGCTTTCCAAATTCTTTGAGTAATC‑AATAGGAGAAAtcaatatgatcgtttctgacatcgaagctaacgccctcttagagagcgtcactaagttccactgcggggttatctacgactactccaccgctgagtacgtaagctaccgtccgagtgacttcggtgcgtatctggatgcgctggaagccgaggttgcacgaggcggtcttattgtgttccacaacggtcaaaagtatgacgttc                                                                                  >  1:114106‑M1/1‑97 (MQ=255)`  
                                                                                                                                                                                                                       `ggCTGACAGTGCATCCACTAAAGTTACCGAGGCTGCCCGTGTGGCAAACCAAGCTCAACAGCTTTCCAAATTCTTTGAGTAATCAAACAGGAGAAAtcaatatgatcgtttctgacatcgaagctaacgccctcttagagagcgtcactaagttccactgcggggttatctacgactactccaccgctgagtacgtaagctaccgtccgagtgacttcggtgcgtatctggatgcgctggaagccgaggttgcacgaggcggtcttattgtgttccacaacggtcacaagtatgacgttcc                                                                                 >  1:736067‑M1/1‑96 (MQ=255)`  
                                                                                                                                                                                                                       `ggCTGACAGTGCATCCACTAAAGTTACCGAGGCTGCCCGTGTGGCAAACCAAGCTCAACAGCTTTCCAAATTCTTTGAGTAATCAAACAGGAGAAAccattatgatcgtttctgacatcgaagctaacgccctcttagagagcgtcactaagttccactgcggggttatctacgactactccaccgctgagtacgtaagctaccgtccgggtgacttcggtgcgtatctggatgcgctggaagccgaggttgcacgaggcggtcttattgtgttccacaacggccacaagtctgacgttcc                                                                                 >  1:215797‑M1/1‑96 (MQ=255)`  
                                                                                                                                                                                                                       `ggCTGACAGTGCATCCACTAAAGTTACCGAGGCTGCCCGTGTGGC‑AACCAAGCTCAACAGCTTTCCAAATTCTTTGAGTAATCAAACAGGAGAAAtcaatatgatcgtttctgacatcgaagctaacgccctcttagagagcgtcactaagttccactgcggggttatctacgactactccaccgctgagtacgtaagctaccgtccgagtgacttcggtgcgtatctggatgcgctggaagccgaggttgcacgaggcggtcttattgtgttccacaacggtcacaagtatgacgttcct                                                                                >  1:534908‑M1/1‑95 (MQ=255)`  
                                                                                                                                                                                                                       `ggCTGACAGTGCATCCACTAAAGTTACCAAGGCTGCCCGTGTGGCAAACCAAGCTCAACAGCTTTCCAAATTCTTTGAGTAATCAAACAGGAGAAAccattatgatcgtttctgacatcgaagctaacgccctcttagagagcgtcactaagttccactgcggggttatctacgactactccaccgctgagtacgtaagctaccgtccgagtgacttcggtgcgtatctggatgcgctggaagccgaggttgcacgaggcggtcttattttgttccacctgtctcttatacacatctccgc                                                                                 >  1:135623‑M1/1‑96 (MQ=255)`  
                                                                                                                                                                                                                             `cAGTGCATCCACTAAAGTTACCGAGGCTGCCCGTGTGGCAAACCAAGCTCAACAGCTTTCCAAATTCTTTGAGTAATCAAACAGGAGAAAtcaatatgatcgtttctgacatcgaagctaacgccctcttagagagcgtcactaagttccactgcggggttatctacgactactccaccgctgagtacgtaagctaccgtccgagtgacttcggtgcgtatctggatgcgctggaagccgaggttgcacgaggcggtcttattgtgttccacaacggtcacaagtatgacgttcctgcatc                                                                           >  1:397835‑M1/1‑90 (MQ=255)`  
                                                                                                                                                                                                                                    `tCCACTAAAGTTACCGAGGCTGCCCGTGTGGCAAACCAAGCTCAACAGCTTTCCAAATTCTTTGAGTAATCAAACAGGAGAAAtcaatatgatcgtttctgacatcgaagctaacgccctcttagagagcgtcactaagttccactgcggggttatctacgactactccaccgctgagtacgtaagctaccgtccgagtgacttcggtgcgtatctggatgcgctggaagccgaggttgcacgaggcggtcttattgtgttccacaacggtcacaagtatgacgttcctgcattgaccaaa                                                                    >  1:460893‑M1/1‑83 (MQ=255)`  
                                                                                                                                                                                                                                     `ccACTAAAGTTACCGAGGCTGCCCGTGTGGCAAACCAAGCTCAACAGCTTTCCAAATTCTTTGAGTAATCAAACAGGAGAAAtcaatatgatcgtttctgacatcgaagctaacgccctcttagagagcgtcactaagttccactgcggggttatctacgactactccaccgctgagtacgtaagctaccgtccgagtgacttcggtgcgtatctggatgcgctggaagccgaggttgcacgaggcggtcttattgtgttccacaacggtcacaagtatgacgttcctgcattgaccaaac                                                                   >  1:644385‑M1/1‑82 (MQ=255)`  
                                                                                                                                                                                                                                      `cACTAAAGTTACCGAGGCTGCCCGTGTGGCAAACCAAGCTCAACAGCTTTCCAAATTCTTTGAGTAATCAAACAGGAGAAAtcaatatgatcgtttctgacatcgaagctaacgccctcttagagagcgtcactaagttccactgcggggttatctacgactactccaccgctgagtacgtaagctaccgtccgagtgacttcggtgcgtatctggatgcgctggaagccgaggttgcacgaggcggtcttattgtgttccacaacggtcacaagtatgacgttcctgcattgaccaaact                                                                  <  1:188676‑M1/301‑221 (MQ=255)`  
                                                                                                                                                                                                                                      `cACTAAAGTTACCGAGGCTGCCCGTGTGGCAAACCAAGCTCAACAGCTTTCCAAATTCTTTGAGTAATCAAACAGGAGAAAccattatgatcgtttctgacatcgaagctaacgccctcttagagagcgtcactaagttccactgctgggttatctacgactactccaccgctgagtacgtaagctaccgtccgagtgacttcggtgcgtatctggatgcgctggaagccgaggttgcacgaggcggtcttattgtgttccacaacggtcacaagtatgacgttcctgcattgaccaaact                                                                  >  1:579799‑M1/1‑81 (MQ=255)`  
                                                                                                                                                                                                                                        `cTAAAGTTACCGAGGCTTCCCGTGTGGCAAACCAAGCTCAACAGCTTTCCAAATTCTTTGAGTAATCAAACAGGAGAAAtcaatatgatcgtttctgacatcgaagctaacgccctcttagagagcgtcactaagttccactgcggggttatctacgactactccaccgctgagtacgtaagctaccgtccgagtgacttcggtgcgtatctggatgcgctggaagccgaggttgcacgaggcggtcttattgtgttccacaacggtcacaagtatgacgttcctgcattgaccaaactgg                                                                >  1:453623‑M1/1‑79 (MQ=255)`  
                                                                                                                                                                                                                                        `cTAAAGTTACCGAGGCTGCCCGTGTGGCAAACCAAGCTCAACAGCTTTCCAAATTCTTTGAGTAATCAAACAGGAGAAAccattatgatcgtttctgacatcgaagctaacgccctcttagagagcgtcactaagttccactgcggggttatctacgactactccaccgctgagtacgtaagctaccgtccgagtgacttcggtgcgtatctggatgcgctggaagccgaggttgcacgaggcggtcttattgtgttccacaacggtcacaagtatgacgttcctgcattgaccaaactgg                                                                >  1:785327‑M1/1‑79 (MQ=255)`  
                                                                                                                                                                                                                                                      `gCTGCCCGTGTGGCAAACCAAGCTCAACAGCTTTCCAAATTCTTTGAGTAATCAAACAGGAGAAAtcaatatgatcgtttctgacatcgaagctaacgccctcttagagagcgtcactaagttccactgcggggttatctacaactactccaccgctgagtacgtaagctaccgtccgagtgacttcggtgcgtatctggatgcgctggaagccgaggttgcacgaggcggtcttattgtgttccacaacggtcacaagtatgacgttcctgcattgaccaaactggcaaagttgcaattg                                                  <  1:565729‑M1/301‑237 (MQ=255)`  
                                                                                                                                                                                                                                                         `gCCCGTGTGGCAAACCAAGCTCAACAGCTTTCCAAATTCTTTGAGTAATCAAACAGGAGAAAtcaatatgatcgtttctgacatcgaagctaacgccctcttagagagcgtcactaagttccactgcggggttatctacgactactccaccgctgagtacgtaagctaccgtccgagtgacttcggtgcgtatctggatgcgctggaagccgaggttgcacgaggcggtcttattgtgttccacaacggtcacaagtatgacgttcctgcattgaccaaactggcaaagctgtctcttata                                               >  1:756398‑M1/1‑62 (MQ=255)`  
                                                                                                                                                                                                                                                                 `ggCCAACCAAGCTCAACAGCTTTCCAAATTCTTTGAGTAATCAAACAGGAGAAAtcaatatgatcgtttctgacatcgaagctaacgccctcttagagagcgtcactaagttccactgcggggttatctacgactactccaccgctgagtacgtaagctaccgtccgagtgacttcggtgcgtatctgaatgcgctggaagccgaggttgcacgaggcggtcttattgtgttccacaacggccacaagtatgacgttcctgcattgaccaaactggcaaagttgcaattgaaccgagagtt                                       >  1:228904‑M1/1‑54 (MQ=255)`  
                                                                                                                                                                                                                                                                      `aCCAAGCTCAACAGCTTTCCAAATTCTTTGAGTAATCAAACAGGAGAAAtcaatatgatcgtttctgacatcgaagctaacgccctcttagagagcgtcactaagttccactgcggggttatctacgactactccaccgctgagtacgtaagctaccgtccgagtgacttcggtgcgtatctggatgcgctggaagccgaggttgcacgaggcggtcttattgtgttacacaacggtcacaagtatgacgttcctgcattgaccaaactggcaaagttgcaattgaaccgagagttccacc                                  >  1:542085‑M1/1‑49 (MQ=255)`  
                                                                                                                                                                                                                                                                       `ccAAGCTCAACAGCTTTCCAAATTCTTTGAGTAATCAAACAGGAGAAAtcaatatgatcgtttcttacatcgaagctaacgcccttttagagagcgtcactacgttccactgcgggtttatctacgactactccaccgatgagtacgtaagctaccgtccgagtgacttcggtgcgtatctggatgcgctggaagccgaggttgcacgaggctgtcttattgtgttccacaacggtcacaagtatgacgttcctgcattgaccaaactggcaatggtgcaattgaaccgagagttccacgt                                 >  1:438236‑M1/1‑48 (MQ=255)`  
                                                                                                                                                                                                                                                                         `gacagcaacagCTTTCCAAATTCTTTGAGTAATCAAACAGGAGAAAtcaatatgatcgtttctgacatcgaagctaacgccctcttagagagcgtcactaagttccactgcggggttatctacgactactccaccgctgagtacgtaagctaccgtccgagtgacttcggtgcgtatctggatgcgctggaagccgaggttgcacgaggcggtcttactgtgttccacaacggtcacaagtatgacgttcctgcattgaccaaactggcaaagttgcaattgaaccgagagttccaccttc                               <  1:653847‑M1/296‑256 (MQ=255)`  
                                                                                                                                                                                                                                                                         `aaGCTCAACAGCTTTCCAAATTCTTTGAGTAATCAAACAGTAGAAAtcaatatgatcgtttctgacatcgaagctaacgccctcttagagagcgtcactaagttccactgcggggttatctacgactactccaccgctgagtacgtaagctaccgtccgagtgacttcggtgcgtatctggatgcgctggaagccgaggttgcacgaggcggtcttattgtgttccacaacggtcacaagtatgacgttcctgcattgaccaaactggcaaagttgcaattgaaccgatagttccaccttc                               >  1:281543‑M1/1‑46 (MQ=255)`  
                                                                                                                                                                                                                                                                                   `gCTTTCCAAATTCTTTGAGTAATCAAACAGGAGAAAtcaatatgatcgtttctgacatcgaagctaacgccctcttagagagcgtcactaagttccactgcggggttatctacgactactccaccgctgagtacgtaagctaccgtccgagtgacttcggtgcgtatctggatgcgctggaagccgaggttgcactaggcggtcttattgtgttccacaacggtcacaagtatgacgttcctgcattgaccaaactggcaaagttgcaattgaaccgagagttccaccttcctcttgagaa                     >  1:52044‑M1/1‑36 (MQ=255)`  
                                                                                                                                                                                                                                                                                    `cTTTCCCAATTCTGTGAGTAATAAACCAGGAGAAAccattatgatcgtttctgacatcgaagctaacgccctcttagagagcgtcactaagttccactgcggggttatctacgactactccaccgctgagtacgtaagctaccgtccgagtgacttcggtgcgtatctggatgcgctggaagccgaggttgcacgaggcggtcttattgtgttccacaacggtcacaagtatgacgttcctgcattgaccaaactggcaaagttgcaattgaaccgagagttccaccttcctcgtgagaac                    <  1:446621‑M1/301‑267 (MQ=255)`  
                                                                                                                                                                                                                                                                                              `tCTTTGAGTAATCAAACAGGAGAAAtcaatatgatcgtttctgacatcgaagctaacgccctcttagagagcgtcactaagttccactgcggggttatctacgactactccaccgctgagtacgtaagctaccgtccgagtgacttcggtgcgtatctggatgcgctggaagccgaggttgcacgaggcggtcttattgtgttccacaacggtcacaagtatgacgttcctgcattgaccaaactggcaaagttgcaattgaaccgagagttccaccttcctcgtgagaactgtattgaca          <  1:778727‑M1/301‑277 (MQ=255)`  
                                                                                                                                                                                                                                                                                                `tttGAGTAATCAAACAGGAGAAAtcaatatgatcgtttctgacatcgaagctaacgccctcttagagagcgtcactaagttccactgcggggttatctacgactactccaccgctgagtacgtaagctaccgtccgagtgacttcggtgcgtatctggatgcgctggaagccgaggttgcacgaggcggtcttattgtgttccacaacggtcacaagtatgacattcctgcattgaccaaactggcaaagttgcaattgaaccgagagttccaccttcctcgtgagaactgtattgacacc        <  1:750359‑M1/301‑279 (MQ=255)`  
                                                                                                                                                                                                                                                                                                `tttGAGTAATCAAACAGGAGAAAtcaatatgatcgtttctgacagcgaagctaacgccctcttagagagcgtcactaagttccactgcggggttatctacgactactccaccgctgagtacgtaagctaccgtccgagtgacttcggtgcgtatctggatgcgctggaagccgaggttgcacgaggcggtcttattgtgttccacaacggtcacaagtatgacgttcctgcattgaccaaactggcaaagttgcaattgaaccgagagttccaccttcctcgtgagaactgtattgacacc        <  1:742876‑M1/301‑279 (MQ=255)`  
                                                                                                                                                                                                                                                                                                  `tGAGTAATCAAACAGGAGAAAtcaatatgatcgtttctgacatcgaagctaacgccctcttagagagcgtcactaagttccactgcggggttatctacgactactccaccgctgagtacgtaagctaccgtccgagtgacttcggtgcgtatctggatgcgctggaagcctaggttgcacgaggtggtcttattgtgttccacaacggtcacaagtatgacgttcctgcattgaccaaactggcaaagttgcaattgaaccgagagttccaccttcctcgtgagaactgtattgacaccct      <  1:127509‑M1/301‑281 (MQ=255)`  
                                                                                                                                                                                                                                                                                                   `gAGTAATCAAACAGGAGAAAtcaatatgatcgtttctgacatcgaagctaacgccctcttagagagcgtcactaagttccactgcggggttatctacgactactccaccgctgagtacgtaagctaccgtccgagtgccttcggtgcgtatctggatgcgctggaagccgaggttgcacgaggcggtcttattgtgttccacaacggtcacaagtatgacgttcctgcattgaccaaactggcaaagttgcaattgaaccgagagttccaccttcctcgtgagaactgtattgacaccctt     >  1:339041‑M1/1‑20 (MQ=255)`  
                                                                                                                                                                                                                                                                                                    `aGTAATCAAACAGGAGAAAgcaatatgatcgtttctgacatcgaagctaacgccctcttagagagcgtcactaagttccactgcggggttatctacgactactccaccgctgagtacgtaagctaccgtccgagtgacttcggtgcgtatctggatgcgctggaagccgaggttgcacgaggcggtcttattgtgttccacaacggtcacaagtatgacgttcctgcattgaccaaactggcaaagttgcaattgaacctagagttccaccttcctcgtgagaactgtattgacactcttg    <  1:235539‑M1/301‑283 (MQ=255)`  
                                                                                                                                                                                                                                                                                                      `aaaacaaacAGGAGAAAtcaatatgatcgtttctgacatcgaagctaacgccctcttagagagcgtcactaagttccactgcggggttatctacgactactccaccgctgagtacgtaagctaccgtccgagtgacttcggtgcgtatctggatgcgctggaagccgaggttgcacgaggcggtcttattgtgttccacaacggtcacaagtatgacgttcctgcattgaccaaactggcaaagttgcaattgaaccgagagttccaccttcctcgtgagaactgtattgacacccttgtg  <  1:107630‑M1/297‑285 (MQ=255)`  

                                                                                                                                                                                                                                                                                                            `|`                                                                                                                                                                                                                                                                                                        
`GGATTCTTGATGACTTTCCAGACGACTACGAGAAGTTTCGCTGGAGAGTCCCATTCTAATACGACTCACTAAAGGAGACACACCATGTTCAAACTGATTAAGAAGTTAGGCCAACTGCTGGTTCGTATGTACAACGTGGAAGCCAAGCGACTGAACGATGAGGCTCGTAAAGAGGCCACACAGTCACGCGCTCTGGCGATTCGCTCCAACGAACTGGCTGACAGTGCATCCACTAAAGTTACCGAGGCTGCCCGTGTGGCAAACCAAGCTCAACAGCTTTCCAAATTCTTTGAGTAATCAAACAGGAGAAACCATTATGTCTAACGTAGCTGAAACTATCCGTCTATCCGATACAGCTGACCAGTGGAACCGTCGAGTCCACATCAACGTTCGCAACGGTAAGGCGACTATGGTTTACCGCTGGAAGGACTCTAAGTCCTCTAAGAATCACACTCAGCGTATGACGTTGACAGATGAGCAAGCACTGCGTCTGGTCAATGCGCTTACCAAAGCTGCCGTGACAGCAATTCATGAAGCTGGTCGCGTCAATGAAGCTATGGCTATCCTCGACAAGATTGATAACTAAGAGTGGTAT  >  NC_001604.1/13268‑13862`

  

Alignment Legend

Aligned base mismatch/match (shaded by quality score): `ATCG/ATCG` `< 3 ≤` `ATCG/ATCG` `< 13 ≤` `ATCG/ATCG` `< 26 ≤` `ATCG/ATCG` `< 36 ≤` `ATCG/ATCG` `< 38 ≤` `ATCG/ATCG`

Unaligned base: `atcg`    Masked matching base: `atcg`    Alignment gap: `‑`    Deleted base: `‑`

Reads not counted as support for junction

read\_name Not counted due to insufficient overlap past the breakpoint.

read\_name Not counted due to not crossing MOB target site duplication.
